# Prognostic accuracy of SIRS criteria and qSOFA score for in-hospital mortality among influenza patients in the emergency department

**DOI:** 10.1186/s12879-020-05102-7

**Published:** 2020-05-29

**Authors:** Sheng-En Chu, Chen-June Seak, Tse-Hsuan Su, Chung-Hsien Chaou, Hsiao-Jung Tseng, Chih-Huang Li

**Affiliations:** 1 Department of Emergency Medicine, Linkou Medical Center, Chang-Gung Memorial Hospital, Taoyuan, Taiwan; 2 Department of Emergency Medicine, Far Eastern Memorial Hospital, New Taipei City, Taiwan; 3 College of Medicine, Chang-Gung University, Taoyuan, Taiwan; 4 Chang Gung Medical Education Research Centre, Chang-Gung Memorial Hospital, Taoyuan, Taiwan; 5 Clinical Trial Center, Chang-Gung Memorial Hospital, Taoyuan, Taiwan; 6 Graduate Institute of Clinical Medical Sciences, College of Medicine, Chang-Gung University, Taoyuan, Taiwan

**Keywords:** “Influenza”, “Mortality”, “Predictors”, “qSOFA”, “Sirs”

## Abstract

**Background:**

The seasonal influenza epidemic is an important public health issue worldwide. Early predictive identification of patients with potentially worse outcome is important in the emergency department (ED). Similarly as with bacterial infection, influenza can cause sepsis. This study was conducted to investigate the effectiveness of the Systemic Inflammatory Response Syndrome (SIRS) criteria and the quick Sequential Organ Failure Assessment (qSOFA) score as prognostic predictors for ED patients with influenza.

**Methods:**

This single-center, retrospective cohort study investigated data that was retrieved from a hospital-based research database. Adult ED patients (age ≥ 18 at admission) with laboratory-proven influenza from 2010 to 2016 were included for data analysis. The initial SIRS and qSOFA scores were both collected. The primary outcome was the utility of each score in the prediction of in-hospital mortality.

**Results:**

For the study period, 3561 patients met the study inclusion criteria. The overall in-hospital mortality was 2.7% (95 patients). When the qSOFA scores were 0, 1, 2, and 3, the percentages of in-hospital mortality were 0.6, 7.2, 15.9, and 25%, respectively. Accordingly, the odds ratios (ORs) were 7.72, 11.92, and 22.46, respectively. The sensitivity and specificity was 24 and 96.2%, respectively, when the qSOFA score was ≥2. However, the SIRS criteria showed no significant associations with the primary outcome. The area under the receiver operating characteristic curve (AUC) was 0.864, which is significantly higher than that with SIRS, where the AUC was 0.786 (*P* < 0.01).

**Conclusions:**

The qSOFA score potentially is a useful prognostic predictor for influenza and could be applied in the ED as a risk stratification tool. However, qSOFA may not be a good screening tool for triage because of its poor sensitivity. The SIRS criteria showed poor predictive performance in influenza for mortality as an outcome. Further research is needed to determine the role of these predictive tools in influenza and in other viral infections.

## Background

Worldwide, influenza has long been a threat to public health [1]. Patients’ outcomes in influenza range from simple upper respiratory infection (URI) to acute lung injury (ALI), acute respiratory distress syndrome (ARDS), and multiorgan dysfunction syndrome. Influenza progresses rapidly and leads to morbidity and mortality within days [2]. Furthermore, influenza increases the mortality of vulnerable patient groups [3]. A modeling study estimated the global influenza-associated respiratory mortality in 33 countries between 1999 and 2015 and showed that the estimated mean annual influenza-associated respiratory excess mortality rate ranged from 0.1 to 6.4, 2.9 to 44.0, and 17.9 to 223.5 per 100,000 individuals in individuals younger than 65 years, between 65 and 74 years, and ≥ 75 years, respectively [4]. A global pandemic of influenza (H1N1) occurred in 2009 and caused more than 18,000 deaths worldwide [5]. Another localized outbreak took place in Taiwan from late 2015 to early 2016, resulting in more than 2000 severe influenza cases (85.5 per million population) and over 160 deaths (6.9 per million) nationwide during this period [6, 7].

Early recognition of patients with potentially worse outcomes is important in the emergency department (ED) [8]. The first clinical prediction rule of influenza infection, comprising 9 parameters, was published in 2004 [9]. In 2011, Oh et al. reported four risk factors associated with disease severity: altered mental status, hypoxia, bilateral lung infiltration, and old age [10]. The geriatric influenza death (GID) score and the Shock Index were recently reported to be prediction rules for patients with influenza [11]. However, not all prediction rules that have been described have provided satisfactory diagnostic strength. Moreover, some laboratory tests in these studies were not routinely checked in the ED and, therefore, they may not be feasible in all EDs. A simple and accurate prediction rule is yet to be developed.

Similar to bacterial infections, the Influenza virus triggers the host immune system and generates a proinflammatory response. A prolonged and excessive inflammatory response results in severe sepsis and poor outcomes [12]. An influenza infection shares many common pathways with the immune response to bacteria and can trigger a similar physiologic inflammatory cascade [12, 13]. Furthermore, influenza could impair antibacterial immune defense mechanisms [14–18] and lead to secondary bacterial sepsis [19, 20]. A multinational multicenter cross-sectional study in Asia found that 4% of all sepsis was caused by influenza viruses [21].

In 1992, the American College of Chest Physicians (ACCP) and Society of Critical Care Medicine (SCCM) Consensus Conference committee introduced the Systemic Inflammatory Response Syndrome (SIRS) criteria to define sepsis and predict in-hospital mortality. The SIRS is a scoring tool that combines data on vital signs and white blood cell (WBC) [8]. In 2016, sepsis was redefined as a “life-threatening organ dysfunction caused by a dysregulated host response to infection” by the Third International Consensus Definitions for Sepsis and Septic Shock (Sepsis-3) [22]. Instead of the SIRS criteria, Sepsis-3 developed the quick Sequential (Sepsis-related) Organ Failure Assessment (qSOFA) score – a simplified version of the SOFA score – as a new sepsis screening tool for use outside the intensive care unit (ICU) [23]. The items included in the qSOFA are readily available in the ED after triage. The performance of the qSOFA score in predicting adverse outcomes has been validated among patients in various clinical settings [24–30]. Most of the results of previous research suggested that the qSOFA score is specific but lacks sensitivity in predicting risk of either mortality or ICU admission [31]. We theorized that the qSOFA score could be a more accurate diagnostic tool compared to SIRS in patients with influenza.

This study was undertaken to evaluate the diagnostic accuracy of the SIRS criteria and qSOFA score in predicting the in-hospital mortality of patients with an influenza infection. An accurate prediction tool will aid early risk stratification and rapid treatment of patients with influenza in the ED.

## Methods

### Study design and setting

This single-center, retrospective cohort study included patients who visited the ED of Linkou Chang Gung Memorial Hospital from 2010 to 2016. The Linkou Chang Gung Memorial Hospital is a tertiary-care medical center in Taiwan with 3406 bed, and, on average, there were approximately 17,000 ED visits per month in 2019. All of the patients’ data were retrieved from the Chang Gung Research Database (CGRD). This large database contains all necessary records from every visiting patient, including details of their vital signs, blood tests, image reports, diagnosis, treatments, and daily medical records of doctors and nursing staff. All data are de-identified and encrypted to protect the participants’ privacy.

Patients with flu-like syndrome in the ED were evaluated by at least one emergency physician and managed by the guidelines approved by our ED committee. Most of these patients received the rapid influenza diagnostic test (RIDT) or influenza reverse transcription-polymerase chain reaction (RT-PCR) test. However, to avoid ED crowding, some patients with minor symptoms would be discharged home directly without further examinations, such as radiographic, laboratory, or flu investigations. This study was approved by the Institute Review Board, Chung Gang Memorial Hospital.

### Selection of participants

Patients aged 18 years or older visiting the ED were enrolled in the analysis if they had a diagnosis of influenza, confirmed by either positive RIDT or RT-PCR test. If patients visited the ED more than once within 30 days with the same diagnosis, they were considered to have the same influenza episode, and data from their last visit would be included in this study.

### Measurements

The vital signs and Glasgow Coma Scale (GCS) score recorded at triage were collated. The qSOFA score ranges from 0 to 3, with one point for each of the following: respiratory rate ≥ 22 breaths per minute, altered mentation, and systolic blood pressure ≤ 100 mmHg. We defined altered mentation as a GCS score < 15 at triage or decreased coma scale compared to the baseline level. A positive qSOFA score was defined as a score of 2 or higher. In addition, we collected SIRS criteria, including body temperature > 38 °C or < 36.0 °C, heart rate > 90 beats per min, respiratory rate ≥ 20 breaths per min, WBC count > 12,000 or < 4000 cells/mm^3^, or > 10% immature (band) forms. The SIRS score ranges from 0 to 4, and a positive SIRS score was defined as 2 or more points.

All the results of initial laboratory tests in the ED were extracted from the electronic medical records; trained hospital personnel who were blinded to the objective of the study calculated the qSOFA and SIRS scores. For patients without blood tests, we defined the WBC score of SIRS as 0, because these patients were relatively few and were discharged after a physician’s assessment. Associated data were obtained, including sex, age, and comorbidities. We used the Charlson Comorbidity Index (CCI) [32] to define the comorbid condition of all patients. Data on all patient outcomes, including ICU admission and in-hospital mortality, were collected.

### Outcomes

The primary outcome of this study was in-hospital mortality, including all deaths that occurred in the ED, ordinary ward, and ICU. The secondary outcome was the ICU admission, comprising two patient groups: one group included patients who were initially hospitalized in the general ward and later transferred to the ICU, and the other group included patients who were directly admitted to the ICU from the ED.

### Statistical analysis

Data from the CGRD were statistically analyzed, and results were presented as means and standard deviations for continuous variables as well as numbers and percentages for categorical variables. Moreover, we calculated the percentages of outcomes by each level of SIRS and qSOFA. Logistic regression models were built to evaluate the predictive ability of these two scoring tools for ICU admission and in-hospital mortality. Akaike’s Information Criterion (AIC) and receiver operating characteristics (ROC) curves with c-statistics were used to compare the predictive performance of the tools. All statistical analyses were undertaken using SAS 9.4 (SAS Institute, Cary, NC, USA) and Stata 14 (StataCorp, College Station, TX, USA). Significance level α was set at 0.05.

## Results

**Fig. 1:**
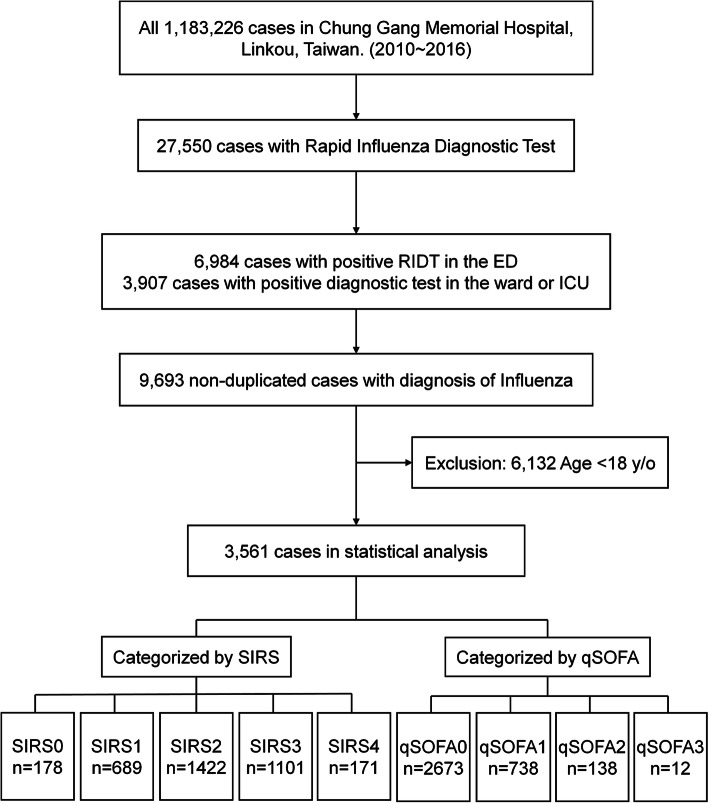
The flow chart of sample selection for analysis

**Fig. 2:**
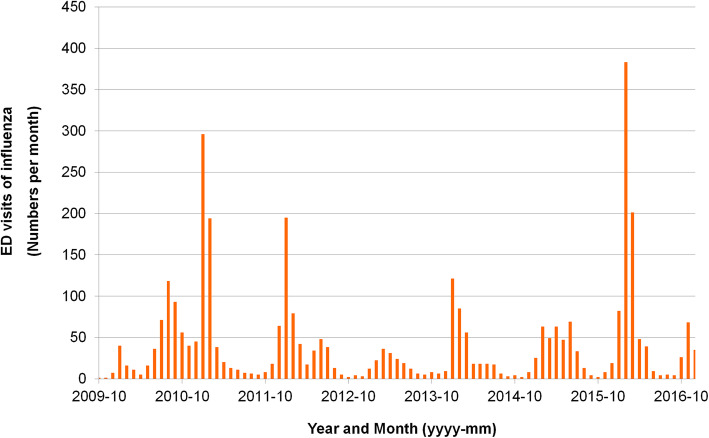
Number of emergency department visits with positive influenza diagnostic test by months

There were 1,183,226 ED visits to Chung Gang Memorial Hospital, Linkou, from January 2010 to December 2016, and the patient disposition flow diagram is shown in Fig. 1. In total, 27,550 patients underwent either RIDT or RT-PCR. Among all of the patients who visited the ED, 9693 patients tested positive for influenza A or B; of these, 3561 were adults and have been included in our analysis. Four major seasonal influenza epidemics were recorded between 2010 and 2016 in Taiwan (Fig. 2) and patient numbers increased accordingly in the CGRD. The largest outbreak occurred in late 2015 and early 2016, with more than 350 visits of adult patients with influenza per month.

**Fig. 3:**
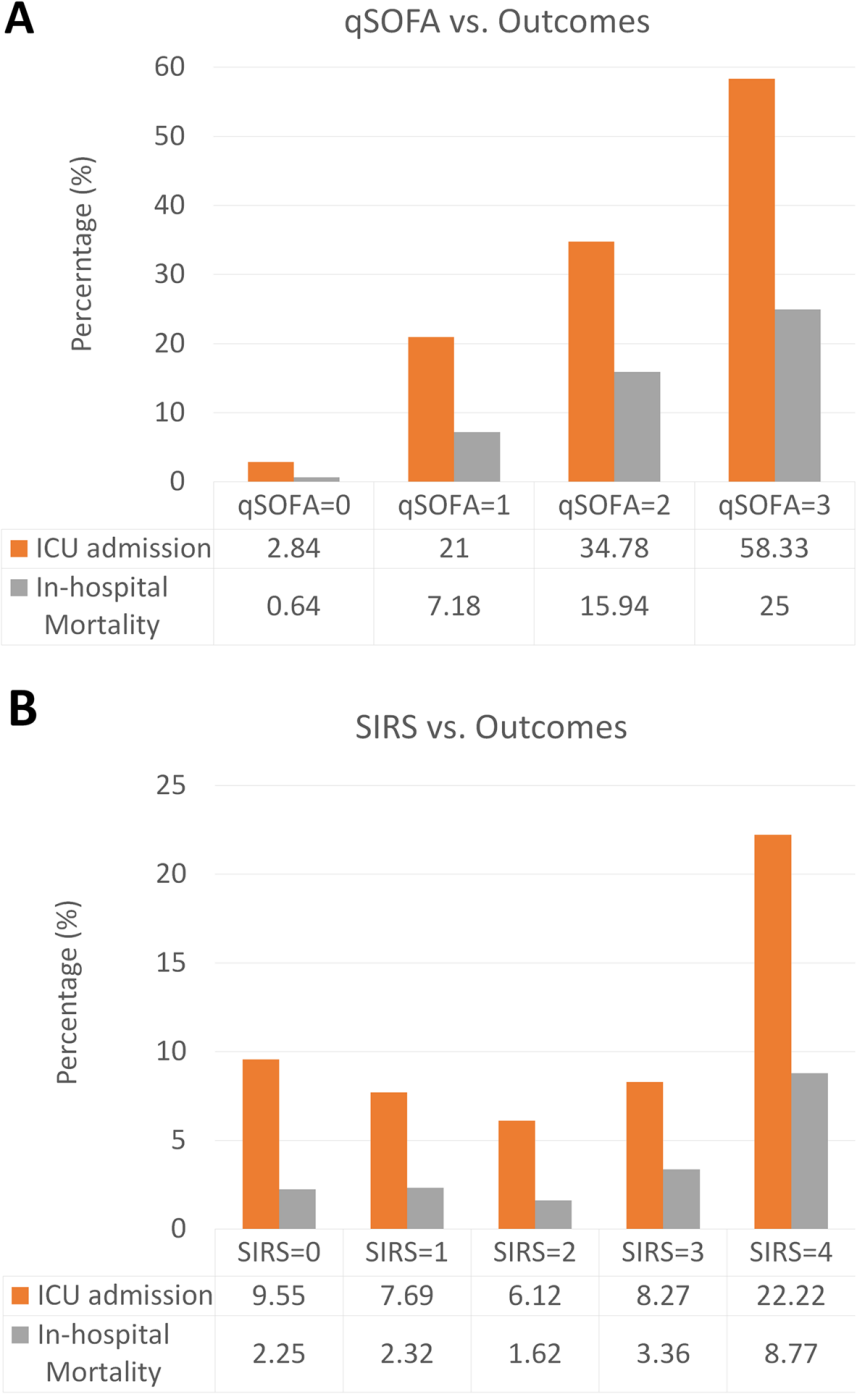
The quick Sequential (Sepsis-related) Organ Failure Assessment (qSOFA) and Systemic Inflammatory Response Syndrome (SIRS) models by outcomes

Baseline patient characteristics are shown in Table 1. The mean age of patients in this study population was 48 years, and 1716 patients (48.9%) were male. The CCI (mean ± SD) was 3.94 ± 4.19. Of the patients with influenza, 1527 (42.9%) were hospitalized (Table 2) and 286 (8.0%) were admitted to the ICU. The overall in-hospital mortality in this study population was 2.7%. Only 31 patients (0.9%) were readmitted within 72 h after direct discharge from the ED. The primary outcome of in-hospital mortality rate was 0.6, 7.2, 15.9, and 25% when the qSOFA score was 0, 1, 2, and 3, respectively. Patients with qSOFA scores of 0, 1, 2, and 3 had ICU admission rates of 2.8, 21, 34.8, and 58.3%, respectively (Fig. 3a). Furthermore, we compared the performance of SIRS criteria for predicting in-hospital mortality, which was not associated with the SIRS criteria based on our results (Fig. 3b).

**Table 1:** Baseline characteristics of patients with influenza

	Mean ± SD / N (%)
Age	48.08 ± 19.51
Sex = male	1716 (48.19)
Vital signs	
Body temperature, °C	38.11 ± 1.17
Heart rate, beats/min	107.64 ± 18.83
Respiratory rate, breaths/min	19.80 ± 3.35
Systolic blood pressure, mmHg	142.03 ± 28.47
Diastolic blood pressure, mmHg	84.06 ± 22.84
Glasgow Coma Scale score	14.65 ± 1.55
White blood cell count (1000/mm^3^)	8.14 ± 4.31
Charlson Comorbidity Index (CCI)	3.94 ± 4.19
Comorbidities	
Myocardial infarction	169 (4.75)
Congestive heart failure	438 (12.3)
Peripheral vascular disease	140 (3.93)
Cerebrovascular disease	491 (13.79)
Dementia	137 (3.85)
Chronic pulmonary disease	960 (26.96)
Connective tissue disease-rheumatic disease	157 (4.41)
Peptic ulcer disease	850 (23.87)
Mild liver disease	706 (19.83)
Diabetes mellitus, without complications	767 (21.54)
Diabetes, with complications	300 (8.42)
Paraplegia and hemiplegia	77 (2.16)
Renal disease	644 (18.08)
Cancer	469 (13.17)
Moderate or severe liver disease	83 (2.33)
Metastatic carcinoma	124 (3.48)
AIDS/HIV	14 (0.39)

*SD* standard deviation, *AIDS* acquired immune deficiency syndrome, *HIV* human immunodeficiency virus

**Table 2:** Distribution of study outcomes

	ED re-admission within 72 h	Hospitalization	ICU admission	In-hospital mortality
All	31 (0.87)	1527 (42.88)	286 (8.03)	95 (2.67)
By SIRS				
0	0 (0.00)	115 (64.61)	17 (9.55)	4 (2.25)
1	5 (0.73)	331 (48.04)	53 (7.69)	16 (2.32)
2	13 (0.91)	562 (39.52)	87 (6.12)	23 (1.62)
3	8 (0.73)	413 (37.51)	91 (8.27)	37 (3.36)
4	5 (2.92)	106 (61.99)	38 (22.22)	15 (8.77)
By qSOFA				
0	19 (0.71)	915 (34.23)	76 (2.84)	17 (0.64)
1	12 (1.63)	492 (66.67)	155 (21.00)	53 (7.18)
2	0 (0.00)	111 (80.43)	48 (34.78)	22 (15.94)
3	0 (0.00)	9 (75.00)	7 (58.33)	3 (25.00)

*ED* emergency department, *ICU* intensive care unit, *SIRS* Systemic Inflammatory Response Syndrome, *qSOFA* quick Sequential Organ Failure Assessment

In-hospital mortality increased significantly in relation to male sex, age, CCI, and qSOFA score (Table 3). The crude OR was 12.1 (95% confidence interval [CI] 6.9–21.0), 29.6 (15.3–57.3), and 52.1 (13–209.3) for qSOFA scores of 1, 2, and 3, respectively, which showed significant differences. The adjusted OR by c-statistics was 7.7 (95% CI 4.4–13.7), 11.9 (5.7–24.8), and 22.5 (4.3–116.3) when the qSOFA score was 1, 2, and 3, respectively.

**Table 3:** Logistic regression models for predicting in-hospital mortality

	Univariate Analysis			Model (SIRS)			Model (qSOFA)		
	OR	p	95% CI	OR	p	95% CI	OR	p	95% CI
Sex (M vs F)	2.93	< 0.001	(1.86–4.62)	2.47	< 0.001	(1.53–3.98)	2.29	0.001	(1.41–3.72)
Age	1.04	< 0.001	(1.03–1.05)	1.03	< 0.001	(1.02–1.05)	1.02	0.01	(1.01–1.04)
CCI	1.13	< 0.001	(1.09–1.18)	0.96	0.262	(0.90–1.03)	0.95	0.167	(0.89–1.02)
SIRS	1.52	< 0.001	(1.21–1.91)						
SIRS 0	1.00			1.00					
1	1.03	0.953	(0.34–3.13)	1.26	0.689	(0.40–3.97)			
2	0.72	0.54	(0.24–2.09)	0.94	0.92	(0.31–2.87)			
3	1.51	0.437	(0.53–4.30)	2.52	0.095	(0.85–7.43)			
4	4.18	0.013	(1.36–12.87)	4.81	0.009	(1.49–15.49)			
qSOFA	4.72	< 0.001	(3.67–6.07)						
qSOFA 0	1.00						1		
1	12.09	< 0.001	(6.96–21.01)				7.72	< 0.001	(4.35–13.70)
2	29.63	< 0.001	(15.32–57.31)				11.92	< 0.001	(5.74–24.77)
3	52.08	< 0.001	(12.96–209.28)				22.46	< 0.001	(4.33–116.61)
Hospital stay	1.04	< 0.001	(1.03–1.05)	1.06	< 0.001	(1.05–1.07)	1.05	< 0.001	(1.04–1.06)
				AIC = 717.991			AIC = 664.974		

*OR* odds ratio, *CI* confidence interval, *AIC* Akaike Information Criterion, *ED* emergency department, *ICU* intensive care unit, *SIRS* Systemic Inflammatory Response Syndrome, *qSOFA* quick Sequential Organ Failure Assessment

**Fig. 4:**
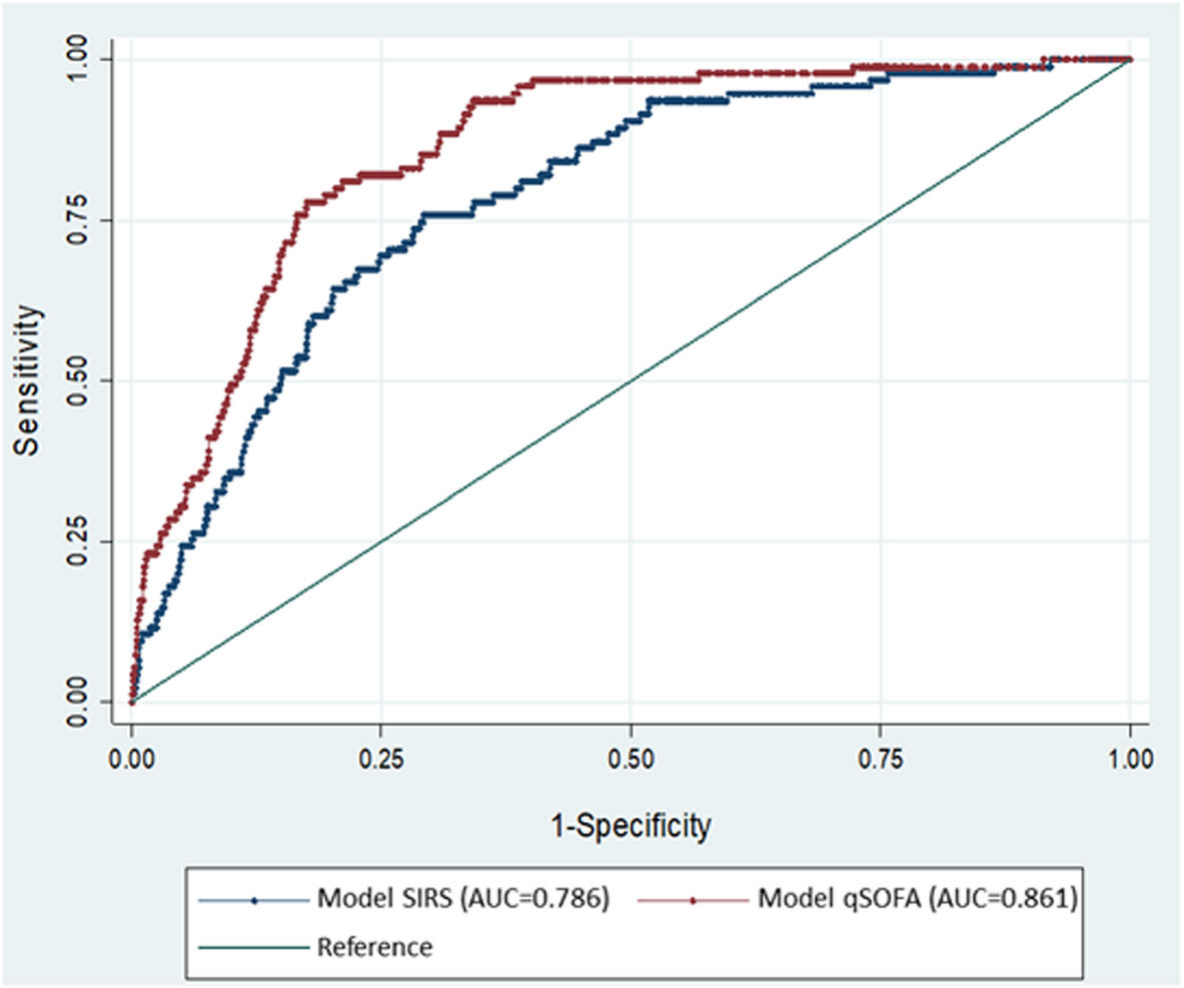
Receiver operating characteristic (ROC) curves of Systemic Inflammatory Response Syndrome (SIRS) model and quick Sequential (Sepsis-related) Organ Failure Assessment (qSOFA) model for predicting in-hospital mortality

The area under the receiver operating characteristic curve (AUC) of the qSOFA model for predicting in-hospital mortality was 0.861, which was significantly higher than the AUC of the SIRS model (0.79; Fig. 4). When the qSOFA score was ≥2, which is the cutoff point defined by Sepsis-3, the qSOFA had high predictive accuracy for ICU admission and in-hospital mortality (94.5 and 94.7%, respectively) although the sensitivity was low (26.3 and 24%, respectively). If the cutoff point was set as a qSOFA score ≥ 1, this would ensure the best Youden Index (0.587 for ICU admission, 0.590 for in-hospital mortality) as well as better sensitivity (82.1 and 82.7%, respectively), which could facilitate clinical decision-making by emergency physicians.

In the SIRS model, when the SIRS score was ≥2 (the cutoff point defined by Sepsis-2), accuracy was merely 25.9 and 25.6% for ICU admission and in-hospital mortality, respectively (Table 4). To obtain the best Youden Index (0.195 for ICU admission, 0.207 for in-hospital mortality), the cutoff point should be a SIRS score ≥ 3. However, the sensitivity, specificity, and likelihood ratio of SIRS ≥3 were poorer than those of qSOFA ≥1. Thus, the qSOFA score is a better tool to predict influenza outcomes.

**Table 4:** Predictive performance of SIRS and qSOFA model in each cutoff point

	Cut-off	ICU admission					In-hospital mortality				
		Sen (%)	Sp (%)	Acc (%)	LR+	LR-	Sen (%)	Sp (%)	Acc (%)	LR+	LR-
SIRS	(≥ 1)	95.8	5	7.4	1.01	0.84	96	5	6.9	0.99	1.21
	(≥ 2)	79	24.4	25.9	1.04	0.86	78.7	24.4	25.6	1.00	1.01
	(≥ 3)	54.7	64.8	64.5	1.56	0.7	56	64.7	64.5	1.29	0.84
	(≥ 4)	15.8	95.5	93.4	3.51	0.88	16	95.4	93.8	3.27	0.90
qSOFA	(≥ 1)	82.1	76.6	76.8	3.51	0.23	82.7	76.3	76.4	3.55	0.34
	(≥ 2)	26.3	96.4	94.5	7.3	0.76	24	96.2	94.7	6.63	0.83
	(≥ 3)	3.2	99.7	97.2	12.16	0.97	1.3	99.7	97.6	16.03	0.98

*Sen* sensitivity, *Sp* specificity, *Acc* accuracy, *LR+* positive likelihood ratio, *LR-* negative likelihood ratio

## Discussion

Worldwide, influenza epidemics emerge almost every year and patient visits to the ED increase dramatically during the flu seasons. Most influenza infections are self-limiting and manageable with symptomatic treatment, and antiviral therapy might not be necessary in most patients. However, during the outbreak in Taiwan in 2015–2016, we observed extremely rapid disease progression in some patients. Severely ill patients may progress to acute hypoxic respiratory failure within 24 h even if the initial chest x-ray shows normal findings. Therefore, a simple early prognostic indicator might be clinically important to an emergency physician. In the present study, we found that the qSOFA score was a better prognostic indicator compared with the SIRS criteria.

Since the qSOFA score was developed, many people have evaluated its clinical value for sepsis. Some articles reported that the qSOFA as a good prognostic predictor [25, 33], whereas other studies found that the qSOFA may not be an adequate screening tool in the ED because of its poor sensitivity [34–36]. A recent meta-analysis showed that the qSOFA was better than the SIRS was in predicting in-hospital mortality of sepsis [37], and a similar finding was observed in our cohort. The predictive performance of the qSOFA was better than that of the SIRS (good accuracy, but poor sensitivity) in the prognostication of patients with influenza, and we rationalized that this finding was related to the septic reaction induced by the influenza or a secondary bacterial infection.

The SIRS criteria have been used to predict outcomes in influenza. Tai et al. conducted a retrospective study that included 409 geriatric ED patients (age ≥ 65 years) who tested positive on RIDT [38] and found that SIRS criteria ≥3 was an acceptable predictor of mortality in this group of patients (OR 3.37, 95% CI 1.05–10.73; sensitivity 60, 95% CI 46–80%; specificity 70, 95% CI 66–75%). The present study included all adult patients with influenza, not merely elderly patients, and we included patients with positive RT-PCR test results for influenza to reduce bias. We found that the SIRS had poor predictivity for outcomes in influenza.

Several previous studies have reported the use of different prognostic scales of pneumonia to evaluate influenza [39–44]. Myles et al. [39] compared the performance of Community Assessment Tools (CATs), CURB-65 score, and the Pandemic Medical Early Warning Score in influenza. They found the CATs were a useful triage tool to predict severe outcomes. However, theirs was a case–control study and was limited to H1N1 infections, which might have conferred some bias. Another retrospective study used eight different scoring tools, including CURB-65, Mortality in Emergency Department Sepsis (MEDS) score, the Nursing Home-Acquired Pneumonia score, PMEWS, Pneumonia Severity Index, severity score for the elderly with community-acquired pneumonia score, SMART-COP Score, and Simple Triage Scoring System, to predict the outcomes of influenza in the ED [40, 43]. These researchers found that the PSI and MEDS scores were moderately predictive of in-hospital mortality, and the SMARTCOP score was a good predictor of ICU admission. In the present study, we did not compare these pneumonia scales because all of these scoring tools need further radiographic or laboratory investigations. We did not routinely arrange these exams for every patient with flu-like symptoms in the ED. In addition, the qSOFA score is much easier and simpler to use for the frontline ED staff. Further prospective studies are required to define the roles of these scoring tools for influenza in the ED.

Other studies have attempted to use serum biomarkers to predict outcomes in patients with influenza. Zimmerman et al. reported that serum levels of C-reactive protein were an early predictor of outcome in the ED [45]. Another report concluded that serum level of lactate dehydrogenase > 600 IU/L was associated with mortality in influenza-induced pneumonia [46]. However, both of the abovementioned studies were limited to H1N1 influenza. Moreover, serum biomarkers were not optimal as early prognostic predictors because we blood tests will not be conducted for every patient with influenza in a busy ED, especially during an epidemic outbreak.

Patel et al. developed a predictive classification tree model to estimate the mortality rates of the human highly pathogenic avian influenza (HPAI) A/H5N1 based on significant predictors of influenza mortality, including age, duration from symptom onset to hospitalization, country, and per capita government health expenditure. However, the quality of data was inconsistent [47]. Those authors included 617 H5N1 cases in their meta-analysis of articles published in any language. There were wide variations in their database, such as with regard to surveillance and clinical care, vaccination policy, lack of data on antiviral treatment, and time from illness onset to the initiation of antiviral treatment. Furthermore, the abovementioned study was limited to HPAI A/H5N1 and did not include all influenza.

Patients with a qSOFA score ≥ 1 might already have clinically apparent illness. Emergency physicians might not always need the qSOFA score to facilitate patient disposition. However, this quantitative scoring tool could generate a more objective and more representative picture of every individual with influenza. In addition, in our data, the CCI, admission rate, and mortality rate were relatively higher than in previous studies. This means that our cohort had more severe illness and was therefore much closely representative of the real ED. We believe that our results are reliable and can be applied in clinical practice, especially in the ED.

### Limitations

This study has strength in numbers, although it was a single-center retrospective study with the inherent limitations of this study design. In addition, we might not have ordered an influenza test for everyone presenting to the ED with fever or URI symptoms, especially in the non-flu season. Moreover, all tests for influenza generate false negatives. These factors might induce some bias. Further studies are required to confirm our findings and prospectively validate the use of qSOFA in this specific patient population.

The definition of “altered mentation” with regard to the qSOFA score has two versions in the literature. One includes a GCS score ≤ 13, which was mentioned in the original study of Sepsis-3 [23]. The other was a GCS score < 15, according to the definitions of sepsis and septic specified by the Third International Consensus [22]. In the present study, we used the GCS score < 15 for analysis. This might have caused an overestimated qSOFA score because of patients with unclear baseline mental status due to underlying disease.

## Conclusions

The qSOFA score is potentially a useful prognostic predictor for influenza and could be applied in the ED after triage, as an early risk stratification tool. Patients with an initial qSOFA score ≥ 1 might need to be hospitalized patients whereas those with a qSOFA score of 3 might need intensive monitoring and aggressive treatment and ICU admission might be indicated. However, the qSOFA may not be a good screening triage tool because of its poor sensitivity in the detection of high-risk patients. The SIRS criteria had poor predictive performance for influenza outcomes. Further studies are needed to determine whether there exists any role for the SIRS criteria and to confirm a role for the qSOFA score in the assessment of patients with influenza as well as other viral respiratory infections.

## Data Availability

The datasets used and analyzed during the current study are available from the corresponding author on reasonable request.

## Abbreviations

ALI: Acute lung injury
ARDS: Acute respiratory distress syndrome
ACCP: American College of Chest Physicians
AIC: Akaike Information Criterion
AUROC: Area under the receiver operating characteristic curve
CGRD: Chang Gung Research Database
CCI: Charlson Comorbidity Index
CI: Confidence interval
CATs: Community assessment tools
CAP: Severity Score for the Elderly with community-acquired pneumonia
ED: Emergency department
GID: Geriatric influenza death
GCS: Glasgow Coma Scale
HPAI: Highly pathogenic avian influenza
ICU: Intensive care unit
LDH: Lactate dehydrogenase
MEDS: Mortality in Emergency Department Sepsis score
NHAP: Nursing Home-Acquired Pneumonia score
PMEWS: Pandemic Medical Early Warning Score
PSI: Pneumonia Severity Index
qSOFA: quick Sequential (Sepsis-related) Organ Failure Assessment
RIDT: Rapid influenza diagnostic test
RT-CPR: Reverse transcription-polymerase chain reaction
ROC: Receiver operating characteristic
SCCM: Society of Critical Care Medicine
STSS: Simple Triage Scoring System
SIRS: Systemic Inflammatory Response Syndrome
URI: Upper respiratory infection
WBC: White blood cell
